# Development and Evaluation of a Therapist Training Program for Psilocybin Therapy for Treatment-Resistant Depression in Clinical Research

**DOI:** 10.3389/fpsyt.2021.586682

**Published:** 2021-02-03

**Authors:** Sara J. Tai, Elizabeth M. Nielson, Molly Lennard-Jones, Riikka-Liisa Johanna Ajantaival, Rachel Winzer, William A. Richards, Frederick Reinholdt, Brian D. Richards, Peter Gasser, Ekaterina Malievskaia

**Affiliations:** ^1^Division of Psychology and Mental Health, The University of Manchester, Manchester, United Kingdom; ^2^Depression Evaluation Services, New York State Psychiatric Institute, New York, NY, United States; ^3^COMPASS Pathways PLC, London, United Kingdom; ^4^Clinical Research Institute, Helsinki University Central Hospital, Helsinki, Finland; ^5^Center for Psychedelic and Consciousness Research, Johns Hopkins Bayview Medical Center, Baltimore, MD, United States; ^6^Aquilino Cancer Center, Maryland Oncology Hematology P.A., Rockville, MD, United States; ^7^Department of Psychological Medicine, Institute of Psychiatry, Psychology & Neuroscience, King's College London, London, United Kingdom; ^8^Sheppard and Enoch Pratt Hospital, Baltimore, MD, United States; ^9^Private Practice, Solothurn, Switzerland

**Keywords:** psilocybin, therapist training, psychological support, treatment resistant, depression, psychedelics, psychdelic therapy, treatment-resistant depression

## Abstract

**Introduction:** Psychological support throughout psilocybin therapy is mandated by regulators as an essential part of ensuring participants' physical and psychological safety. There is an increased need for specially trained therapists who can provide high-quality care to participants in clinical studies. This paper describes the development and practical implementation of a therapist training program of psychological support within a current phase IIb international, multicenter, randomized controlled study of psilocybin therapy for people experiencing treatment-resistant depression.

**Description of Training Program:** This new and manualized approach, based on current evidence-based psychotherapeutic approaches, was developed in partnership with different mental health researchers, practitioners, and experts; and has been approved by the FDA. Training consists of four components: an online learning platform; in-person training; applied clinical training; and ongoing individual mentoring and participation in webinars.This paper provides a brief overview of the method of support, the rationale and methodology of the training program, and describes each stage of training. The design and implementation of fidelity procedures are also outlined.

**Lessons Learned:** As part of the phase IIb study of psilocybin therapy for treatment-resistant depression, 65 health care professionals have been fully trained as therapists and assisting therapists, across the US, Canada and Europe. Therapists provided informal feedback on the training program. Feedback indicates that the didactic and experiential interactive learning, delivered through a combination of online and in-person teaching, helped therapists build conceptual understanding and skill development in the therapeutic approach. Clinical training and engagement in participant care, under the guidance of experienced therapists, were considered the most beneficial and challenging aspects of the training.

**Conclusions:** Clinical training for therapists is essential for ensuring consistently high-quality psilocybin therapy. Development of a rigorous, effective and scalable training methodology has been possible through a process of early, active and ongoing collaborations between mental health experts. To maximize impact and meet phase III and post-approval need, enhanced online learning and establishing pathways for clinical training are identified as critical points for quality assurance. This will require close public, academic and industry collaboration.

## Introduction

Increasing interest in the therapeutic potential of psychedelic compounds for a range of mental health conditions highlights the need for training therapists in supporting participants in clinical studies. This paper describes the rationale, methodology and key learnings from a therapist training program for a phase IIb international, multicenter, randomized controlled study of psilocybin therapy in participants with treatment-resistant depression (TRD); clinical trial number NCT03775200.

Psilocybin is a molecule present in over 200 species of psychoactive mushrooms. It has a dose-dependent capacity to facilitate the experience of non-ordinary states of consciousness, and together with compounds like lysergic acid diethylamide (LSD), mescaline, and N,N-Dimethyltryptamine (DMT), is part of a group of drugs called psychedelics. Psilocybin is a partial-agonist of the 5HT group of receptors, including the 5HT_2A_ receptor subtype. The activation of 5HT_2AR_ results in subjective alterations of perception, mood and cognition during the acute effects of psilocybin. This has potential benefits for mood disorders, as perception and cognition appear to become more flexible, enabling opportunities for new perspectives and insights to be generated, potentially leading to new and novel solutions for ongoing psychological distress (1). Psilocybin effects generally peak around 90 min after ingestion, then gradually subside and resolve in 4–6 h.

Psilocybin and other psychedelic compounds have attracted attention from researchers and clinicians for their potential to catalyze therapeutic change, when taken within a therapeutic setting, in people diagnosed with depression (1), obsessive-compulsive disorder (2), alcohol dependence (3), nicotine dependence (4), and anxiety associated with cancer (5–7). The effects of psychedelics in general, and specifically psilocybin, for other problems are currently being investigated in several pilot studies. Early research with psilocybin has shown signals of immediate, significant and often enduring clinical improvements in depression and anxiety. Such effects are thought to result from a combination of the psychopharmacological effects of psilocybin and the participants' subjective experiences, including generation of insights and subsequent changes in cognition and behavior (8, 9).

Administration of psilocybin in a research setting requires approval from regulators, such as the FDA and United States Drug Enforcement Administration, in addition to approval from an Institutional Review Board or Research Ethics Committee. Just as regulators require consistent data about the synthesis, purity and stability of a specific formulation of psilocybin used in the clinical development program, they similarly require the shared consistency of another equally important component of the treatment approach - the psychological support. To address the need for participant safety and consistency in the delivery of the psychological support, we have created and implemented a therapist training program for the phase IIb clinical trial of psilocybin therapy for TRD.

The study aims to determine the optimal single dose of psilocybin, administered with psychological support, in a clinical setting. The 216 participants are randomized to low (1 mg), medium (10 mg) or high (25 mg) doses of COMP360, a high-purity polymorphic crystalline formulation of psilocybin produced under Good Manufacturing Practice, administered in conjunction with psychological support by specially trained therapists. The study design has been agreed with the European Medicines Agency, FDA, and approved by regulators in 10 countries in North America and Europe. Therapists' professional qualifications, as well as their training format and content, were approved by the FDA and included in the Investigational New Drug program. In 2018, the FDA granted a Breakthrough Therapy designation to COMP360 for TRD. The program of research is managed by COMPASS Pathways PLC., a mental health care company dedicated to accelerating patient access to evidence-based innovation in mental health.

## Description of Training Program

### Therapeutic Model

The principles of psychological support in the current trial, to be outlined in a separate publication, are informed by several factors:

- An empirical theoretical framework based on Perceptual Control Theory (10) that integrates validated evidence-based psychotherapeutic theories and approaches common to therapies such as Cognitive Behavioral Therapy (11), Mindfulness-based Therapy (12), Method of Levels (13), Acceptance and Commitment Therapy (14), and Focusing (15);
- Extensive historic experience in the use of psilocybin in research and clinical settings (7, 16, 17);
- Current understanding of the psychopharmacology and mechanism of action of psilocybin (18–21);
- Regulatory and payor perspectives on the clinical development of psilocybin therapy.

Perceptual Control Theory (PCT) (10) informed the current approach as it offers a biopsychosocial framework for understanding and integrating key principles evident across the general literature on psychological functioning and the psilocybin literature. PCT proposes that human health is dependent on effective homeostatic control of important biological, social, and psychological variables, through feedback. This has particular relevance for TRD, where people's difficulties in controlling variables such as attention and negative thinking act as maintaining factors (11). From a PCT perspective, when we develop awareness of the internal variables we are controlling, we can develop solutions to balancing incompatible variables. As an example from psilocybin therapy, a client experiencing depression described holding two conflicting standards; wanting meaningful intimate relationships whilst also wanting to avoid vulnerability. As psilocybin therapy potentiated changes in usual patterns of perception, mood and cognition, it brought new opportunities for reorganizing and balancing such psychological variables (22). Therapists' training incorporates a basic overview of this theoretical framework to inform practice; describing how participants might develop awareness of personal goals through increased attentional mobility, introspection, and problem solving. This informs therapists' patient-centered and non-directive practice, utilizing a questioning style which supports participants in generating their own potential solutions for resolving incompatible goals, rather than teaching, offering advice, or making assumptions. These principles remain consistent with core therapist skills identified in other psychedelic interventions (23), meaning that there is considerable similarity between the current approach and support in other psilocybin therapies. These principles also informed item development in a fidelity scale, as described below in the section “Ensuring Consistency.”

The primary purpose of psychological support throughout is to ensure participants' physical and psychological safety and minimize the number of any potential adverse events. The most common adverse effect in psilocybin sessions is mild-to-moderate transient anxiety that could potentially lead to more intense or longer-lasting anxiety and paranoia. Extended periods of overwhelming emotional arousal are not only distressing to participants, but could decrease positive treatment outcomes (24). Skillful psychological support helps participants engage with all aspects of their experiences, while keeping more challenging emotions within the participant's bandwidth of tolerance, whereby emotions are not so prolonged and overwhelming that they are unproductive, thus facilitating a sense of safety. To do this, two key principles - *self-directed enquiry* and *experiential processing* - are introduced to and practiced with participants during preparation.

#### Self-Directed Enquiry

A key goal for therapists is to help participants direct attention toward internal experiences as they emerge in the present moment, which may involve noticing foreground and background thoughts, emotions, physical sensations, images and memories. The aim is to bring experiences into awareness and potentially consider them from different perspectives, a key process within most psychological interventions (25), to increase psychological flexibility, introspection and problem solving (11–15, 26). This is described as “self-directed enquiry.” To facilitate this process, therapists use sensitive but searching questions to help participants mobilize their attention to experiences as they arise. Therapists demonstrate and practice self-directed enquiry with participants at all stages of the therapeutic process. If practiced sufficiently during preparation, self-directed enquiry could be particularly helpful should challenging experiences, such as anxiety-inducing material, emerge during a psilocybin session.

#### Experiential Processing

Experiential processing refers to the sustaining of attention on an experience in the present moment so it can be processed in a meaningful way. Awareness of and re-evaluation of important biological, social, and psychological variables can potentiate new insights and facilitate change (10, 13). The importance of experiential “exposure” is well documented throughout the literature (26) Experiential processing thus requires a willingness to stay with thoughts, feelings, sensations or emotions until they have passed or evolved. This is expressed as moving “in and through” (27).

### Overview of the Therapeutic Process

Psilocybin therapy has three phases: *preparation, psilocybin session*, and *integration*. Safety is a foundation for all phases. The therapeutic focus is different at each phase.

Preparation focuses on establishing the therapeutic alliance between the therapist and participant, and demonstrating and practicing self-directed enquiry and experiential processing. Therapeutic alliance develops when the therapist is genuinely curious and cultivates trust through focused presence; considered one of the core elements in ensuring a safe and meaningful psilocybin session.

During preparation, the therapist aims to build the participant's capacity to navigate distressing experiences through self-directed enquiry and experiential processing, using topics chosen by the participant. Practicing this approach during preparation helps the participant engage with their experiences in the psilocybin session, including physical and psychological challenges.

During the psilocybin session, the focus is to ensure psychological distress is not overwhelming, in order to facilitate a meaningful experience. The participant is encouraged to maintain a courageous, trusting and open attitude in order to move “in and through” the full range of internal experiences. Therapists are encouraged to maintain awareness of their own experiences during the session, including boredom, anxiety and fear, while modeling the same attitudes of openness and interest toward participants.

The therapeutic focus of integration is to reflect on the experience and generate insights following the psilocybin session. Participants are encouraged to describe and connect with the range of emotional, cognitive and physical experiences of the psilocybin session and relate them to their personal narrative. The integration process will generally unfold over time as the participant begins to implement changes in their life based on the experiences and insights of their psilocybin session.

### Therapist Qualifications, Background, Experience, and Selection Criteria

All therapists in the clinical development program of psilocybin therapy for TRD are required to be mental health care practitioners with a professional license in good standing. They must have demonstrated clinical experience in areas required for psychotherapy or mental health counseling. The FDA currently requires all United States therapists to have at least a Masters' level of education and that two trained therapists are present for all psilocybin sessions. The lead therapist on each treating team must be fully trained and the co-therapist is able to gain their necessary clinical training alongside the lead therapist during the study. Although it is not mandatory for one of the therapist dyad to be a psychiatrist, a psychiatrist must, however, be present on premises near to the psilocybin session to respond to emergencies. Psychiatrists in this role must complete specific training, separate from the therapists' training.

Therapists for the phase IIb study were selected by study sites according to country, state, and institution-specific requirements, and interviewed by members of the COMPASS training team. Emphasis was placed on exploring therapists' motivations for getting involved in the study; their essential qualities of presence, openness and patience; and their familiarity with principles of Good Clinical Practice (GCP)-compliant research. We sought therapists with clinical experience and a demonstrated ability to care for people experiencing severe psychological distress, as these skills were likely to translate well into maintaining calm and reassuring presence while caring for participants during a psilocybin session.

At the time of writing, more than 65 therapists and assisting therapists had been fully trained to work across several studies within the COMPASS clinical development program of psilocybin therapy for TRD in North America and Europe. The therapists currently working on the phase IIb study includes predominantly psychologists, as well as psychiatrists, masters level practitioners, nurses, diploma level CBT therapists and PhD mental health specialists. Years of experience since qualification averaged 25.9 years (SD 8.8) with a range of 2–32 years. Therapists reported clinical experience in a number of areas (some more than one), including adult mental health; addictions; dementia; physical health; child/developmental; family therapy, and eating disorders.

### Training Program Structure

Development and piloting of the training was overseen by a team with expertise and experience in therapeutic and research applications of psilocybin, research and training methodology in psychological treatments, and extensive clinical experience of people with TRD. The goal was to engage advisors and trainers with sufficient clinical experience in conducting psilocybin research sessions, which among our trainers, ranged between 25 and 300+ psychedelic research sessions. Having an “expert through experience” on the team, who had previously participated in a clinical study of psilocybin for TRD, firmly oriented the training processes around participant experience and needs. The expert group approach was applied to every stage of the training process, from therapist selection to mentoring and skill maintenance during the active stage of clinical trials.

The therapist training program for the phase IIb study entails four components: (1) an online learning platform, (2) in-person training, (3) clinical training, and (4) ongoing individual mentoring and webinars. All therapists are required to complete steps 1–3 of the training before they can lead sessions independently, and to engage in step 4 to continue their professional development.

#### Step 1. Self-Paced Online Learning

An interactive online platform was developed, consisting of over 20 hours of video modules and training materials covering a general overview of psilocybin research and information about the therapeutic approach. Information contains essential, but not exhaustive, resources on the neuroscience and psychopharmacology of psilocybin; medical, psychological and ethical considerations of psilocybin research; and privacy, confidentiality and GCP. Training modules on the therapeutic approach focus on practicalities of each stage of the therapeutic process, including video re-enactments of clinical scenarios, a therapy manual with study-specific appendices, and Supplementary Materials such as slides, academic papers, adherence rating scales, and other resources.

As all subsequent training steps are interactive, requiring a thorough knowledge of the principles and methods, therapists cannot progress before completing the online training. The platform continues to play an important role in professional development, even after therapists begin leading psilocybin sessions independently. The platform was named “The Shared Knowledge Platform” to emphasize the importance of continuous learning and incorporating clinically-relevant insights from study therapists in a timely manner. Although the core principles of the therapeutic approach remain the same, the knowledge and feedback from study therapists are integral to the optimization of therapeutic care delivery and participant safety.

#### Step 2. Interactive 5-Day In-person Training

The purpose of interactive training is for therapists to translate their learning from the online training modules into practice and demonstrate their understanding of core principles and methods of psychological support throughout all stages of psilocybin therapy. Training is conducted in groups of 10–15 people, led by experienced therapists and trainers. Most of the time is spent in role-plays of short clinical scenarios, anonymized and adapted from previous or current studies. Role-plays based on examples from therapists' own lives, provide first-hand experience of self-directed enquiry and experiential processing. During in-person training, emphasis is placed on providing feedback from peers and trainers. Peer interactions are particularly important as they engage the entire group and enable the trainer to assess therapists' understanding of the approach, and their interpersonal and communication skills as well.

On rare occasions, trainers might have concerns about a therapist's conduct or there is evidence of poor understanding of the core principles of psychological support which could prevent a therapist from being effective. If the conduct or understanding cannot be addressed through feedback or additional training, it is recommended that the study site engage another therapist. In-person training provides opportunities to assess the skills and areas of focus for additional training, and also build a community of competent, motivated therapists who support each other in building a body of mutual knowledge.

#### Step 3. Clinical Training

As agreed with the FDA, all therapists must gain clinical experience in at least four different psilocybin research sessions before leading psilocybin sessions independently. Most therapists in phase IIb gained their clinical experience by supporting participants in our phase I study: COMP002 – The Effects of Psilocybin on Cognitive and Emotional Function in Healthy Participants. This requirement is waived, in part or in full, for therapists with prior experience in supporting people in 3,4 methylenedioxy-methamphetamine (MDMA), LSD, or other psilocybin trials.

To build further capacity at active sites and mitigate potential turnover of therapists, therapists-in-training are now attending the psilocybin sessions in the current phase IIb study, as assisting therapists, alongside more experienced therapists, to gain clinical experience. Clinical training is always supervised by experienced therapists, and used as an opportunity to deepen therapists' understanding of core principles and to build their confidence.

After completing steps 1–3, therapists are eligible to lead psilocybin therapy sessions independently. All therapists are expected to engage in mentoring sessions at least once a month and to engage in continuous professional development.

#### Step 4. Continuing Professional Development

##### Mentoring

The term “mentoring” is closely related to supervision practices common in psychotherapy training (28). The word “mentor” is used instead of “supervisor” as mentors in the phase IIb study do not have a formal managing, evaluating or enforcing role in relation to their mentees, nor do mentees work under their mentors' licenses. Otherwise, the content of mentoring is similar to that of clinical supervision.

The goal of mentoring is to develop and maintain strong professional skills and capabilities as a psilocybin therapist, and to ensure the fidelity of the treatment approach within the study context. Mentoring is not personal psychotherapy, nor is it a form of, or substitute for, fundamental clinical training or line management.

A common structure is followed in mentoring meetings, as recommended in evidence-based mentoring practices (28). Study-specific mentoring guidelines were developed by the mentoring team during a 3-day working group meeting. Guidelines are provided to all mentees at the outset, and the mentoring team meets monthly for “peer mentoring” to ensure consistent delivery of mentoring and resolve any emerging challenges. Mentoring sessions are structured around discussions of clinical scenarios and lessons to be learned from supporting participants in the study. Mentors share fidelity ratings and deliver feedback, along with discussing areas for improvement and potential protocol deviations, with the objective of ensuring consistency and therapist skill development. After each meeting, the mentor and mentee formulate key “take-home messages” and specific action points together, and provide a written summary.

##### Webinars

Therapists are required to participate in at least 50% of the professional development webinars usually conducted monthly. In these webinars, therapists present clinical case studies to their peers and trainers. Discussion content and key learning points are recorded and shared with therapists for future reference. Based on therapists' requests and learning needs that emerge during the study, some of the webinars also concentrate on specific themes, such as integration practices. Webinars aim to address emerging questions and issues in a timely manner and sustain and improve consistency across sites in the study. As different sites recruit at different rates, group webinars create an environment in which to share best clinical practices and maintain therapists' skills.

### Additional Considerations

Several additional considerations regarding the specific challenges of a multisite clinical trial with a psychedelic compound are important to the training program.

#### Ensuring Consistency

Fidelity to the therapeutic approach ensures good internal validity, increases the replicability of an intervention, is likely to lead to better outcomes and participant care, and facilitates further therapy optimization research (29). Demonstrated fidelity to the approach is a regulatory requirement for research and will subsequently determine whether a course of treatment is reimbursed post-approval.

To assess consistency, fidelity scales specific to the intervention were developed to quantify adherence to the therapeutic approach in the context of research. In this study, the fidelity rating scale comprises items related to general safety and GCP, and items that assess principles and methods of the therapeutic approach, as outlined in the psilocybin therapy manual. These include key competencies and actions required of therapists during preparation (25 items), session (18 items) and integration (12 items). Example items for preparation include how well the therapist, “created and communicated a setting of safety and support”; for session, how they “encouraged and facilitated the participant attending to inner experiences and bodily sensations (including challenging experiences and sensations) balanced with periods of communication and movement, where necessary”; and for integration, “facilitated the participant talking freely about the psilocybin session, using active listening and curious questioning” (integration). Therapists' fidelity is rated against criteria for core competencies, on a three-point scale of “*Yes”*; “*Yes, but below the desired level”*; or “*No.”* The current version of the fidelity scale is in the process of ongoing validation and refinement, and will be made available in a separate publication.

At the time of writing, fidelity raters in our phase IIb study are study trainers or therapists with clinical experience, or trained raters with no clinical experience. Video-recorded therapy sessions are used to assess fidelity. Non-therapist raters are required to complete online modules and attend at least three group training sessions, including the analysis and rating of standard clinical scenarios, followed by group discussion led by experienced trainers.

Across the study, all therapists are asked to use the fidelity scale to self-rate every session they conduct. For the purpose of training, such self-rating reports are collated with the fidelity ratings completed by raters and discussed at mentoring meetings.

#### Safety Considerations

Ensuring consistent ethical behavior of therapists is vitally important in psychedelic therapy and research. Participants might be more suggestible, and therefore more vulnerable, during a psychedelic therapy session (30). We ask all study therapists to commit to a therapists' code of ethics, placing the participant's well-being above all, and setting aside personal, ideological, religious or spiritual convictions. Trainers observe therapists during in-person and clinical training, and ongoing mentoring, in order to identify and address any attitudes or behaviors that could place vulnerable participants at risk for psychological manipulation or other unethical behavior.

#### Cultural Sensitivity

For the phase IIb trial, we have established 21 sites in 10 countries across North America and Europe. Given the prevalence of TRD across cultures and socio-economic structures, developing culturally informed approaches to psilocybin therapy and training is paramount. We have actively engaged with participants and experts, trial authorities, culturally diverse study teams and therapists to help identify and address potential challenges and create opportunities for ensuring cultural sensitivity.

We have encountered the challenge of therapists conducting therapy in their national language, which is not the same across all sites. To mitigate potential language discrepancies, a shared language document has been created to guide the selection of suitable translations for words or phrases that do not directly translate from English to the destination language, such as German, Dutch, Danish, Portuguese and Spanish. Therapists are asked to use suitable translations consistently. When possible, therapists are assigned a mentor who speaks the language in which they are conducting the trial, to ensure a thorough understanding of concepts and practices.

The implications of cultural variation on the efficacy of training, and the consistency and quality of support, are not yet clear. We recognize the need for understanding and training in cultural sensitivity, especially when interventions based on interpersonal trust are delivered. We also recognize that cultural sensitivity is not limited to nationalities or languages, but extends to socio-economic background and social determinants of health, including the ability to access quality care, and we plan to introduce a new module on cultural competency in the future.

## Lessons Learned From Therapist Feedback

To develop and improve the training program, all therapists were invited to complete a short anonymous survey after completing the training, and 37 responded.

Therapists rated the quality of the training overall, as well as each section of training (online manual, online videos, face-to-face training, and clinical experience). Ratings were on a Likert scale from 1 to 5 [poor (1), fair (2), average (3), very good (4), excellent (5)]. All therapists rated the training overall as very good (*N* = 14) or excellent (*N* = 23); in-person training was rated as very good (*N* = 13) or excellent (*N* = 24); the online therapy manual was rated as average (*N* = 2), very good (*N* = 13) and excellent (*N* = 22); and the online demonstration videos were regarded as average (*N* = 1), very good (*N* = 12) and excellent (*N* = 24).

Therapists also reported whether the feedback they received from trainers was sufficient, using a 3-point Likert scale (1 = Insufficient, 2 = Sufficient, 3 = More than enough); of which 18 rated “sufficient” and 19 “more than enough.” For ratings of how useful the feedback was, on a 3-point scale (1 = not useful, 2 = somewhat useful, 3 = very useful), 2 reported feedback as “somewhat” and 34 reported it as “very” useful.

Therapists also provided qualitative feedback about what they had found most and least useful, challenging, and whether they felt ready to work with participants. After 35% of participants in the phase IIb trial had received psilocybin therapy, we conducted 90-min interviews with 11 therapists at high-recruiting sites to explore the same questions.

Qualitative data from the surveys and interviews were not subject to formal analysis, least of all because there was little variation in what was reported.

All therapists commented that the training was comprehensive and well-structured, and prepared them well for seeing participants in the study. They reported that the online Shared Knowledge Platform served as a good resource before and during the study, and even after the clinical training was completed. Based on this feedback, we plan to expand the clinical content of the online training to further support therapists who are actively working with participants in the trial. Specifically, additional modules on the implications and support for psychiatric and medical co-morbidities, suicidality, and medication washout were valued resources and might further improve participant safety. When the current approach is used in other studies, additional information on specific diagnostic categories should be included in the platform. We also plan to expand the modules on cultural competencies, and self-care resources for therapists, as an essential part of training.

All therapists reported that the 5 days of initial in-person training was sufficient. They also commented that to fully benefit from in-person training, it was important first to have familiarized themselves with the principles of the approach and methods for the study, as outlined on the Shared Knowledge Platform. To ensure this is the case, we plan to introduce short quizzes and other forms of assessment to monitor engagement and understanding of the materials on the platform.

Therapists also commented that while didactic and experiential interactive training help with conceptual understanding and skill development in the therapeutic approach, clinical training and engagement in participant care during psilocybin therapy research sessions are particularly beneficial, although challenging.

Feedback from the survey and interviews has limitations. First, the study is in its early stages and most therapists have supported only a small number of participants. Second, given the limitations of the study design, we are not able to examine any potential relationship between therapists' training experience and the safety and efficacy outcomes of their participants.

## Discussion

The training of clinicians for clinical work with psychedelics has not yet been a subject of formal inquiry or research. Related programs run by research centers or sponsors of psychedelic drugs progressing through the FDA drug development process are drug, indication, and trial specific^1^. Outside clinical trials, some postgraduate certificate programs offer training for licensed therapists wishing to add education and skills related to psychedelic medicines to their professional development, but do not directly or fully authorize graduates to participate as research therapists^2^^,^ ^3^. Although other programs are related in their focus on the clinical applications of psychedelics, they are not specific to the COMPASS protocol, and are not a substitute for the training program described herein.

Another salient question in the field of psychedelic therapy training is the need for, or relevance, of a therapist's personal experience of the study drug. Some advocate for the inclusion of such experiences in training programs (23), and others have cautioned that the decision to have and discuss such experiences requires careful forethought by clinicians (31). No research has yet demonstrated the impact of therapists' training, or other kinds of personal experience, with psychedelics on clinical outcomes, and the inclusion of such experiences may be a barrier to the inclusion of a diverse group of therapists, place trainers and trainees in dual roles, and even stigmatize those who choose to pursue psychedelic-assisted therapy as a professional. Still, there is anecdotal evidence that some therapists find some personal experiences to be helpful in their professional development [e.g., Halberstadt (32)]. The current program does not include opportunities for personal experience of psychedelics yet respects and allows for discussion of therapists' experiences during training.

Clinical training is critical in ensuring high-quality psilocybin therapy in the context of clinical trials. While didactic training could be outsourced to academic institutions and experienced private therapists, clinical training can only be conducted at selected research sites that have the capacity for a consistently high enrolment and the availability of experienced therapists motivated to train and mentor new therapists. Focusing on selected academic and clinical partners as a base for clinical training will ensure a consistent process of certification of therapists-in-training, provide trainees with continuous educational and professional development credits, and establish training centers of excellence involved in clinical research and care delivery.

Ongoing mentoring and professional development activities, although straightforward, could be logistically challenging at present, given that therapists span time zones from California to Eastern Europe. With increasing numbers of trainees and psilocybin studies consolidating in selected academic centers, these challenges can potentially be resolved with more regional training centers and therapist communities. This consideration leads us to focus on a “train the trainer” program, in which selected experienced therapists undergo additional training to be able to lead future training events and programs.

The design and implementation of fidelity procedures are essential and require a dedicated team with a specific set of skills. We have learned from experience that introducing fidelity assessments early in the training process is essential to guide the direction of training and consolidate the learning for therapists. For a phase III study, we plan to provide raters with a specially designed fidelity rating manual that would be an essential part of training for fidelity raters. In addition, we are investigating the value of machine learning and artificial intelligence to improve fidelity measures as well as the quality of training and feedback.

## Conclusion

Training for psilocybin therapists is essential to ensure consistent and high-quality care for participants in clinical studies and potential post-approval applications. Early, active and ongoing collaborations between mental health experts is essential for the continuous development of a training methodology that is rigorous, effective and scalable. To maximize impact and to meet phase III and post-approval need, we consider the adoption of technology for enhanced online learning and the establishment of pathways for clinical training to be critical points for quality assurance. These goals can only be achieved through close public, academic and industry collaborations united by the common goal of transforming mental health care.

## Data Availability Statement

The raw data supporting the conclusions of this article will be made available by the authors, without undue reservation.

## Ethics Statement

Ethical approval was not provided for this study on human participants because the research involved a survey of training participants that was conducted as part of an internal educational program evaluation process and therefore not requiring IRB. Written informed consent for participation was not required for this study in accordance with the national legislation and the institutional requirements.

## Author Contributions

ST led the development of the psychological approach and therapeutic model, and the design and content development of the therapist training program, including mentoring and fidelity procedures and delivered the majority of the therapist in-person training, and contributed to the writing of this manuscript. EN provided some of the therapist in-person training, contributed to the development of the mentoring and fidelity procedures, and contributed to the writing of the manuscript. PG, WR, BR, R-LJ, and FR contributed to the development of the therapeutic model, therapist recruitment, training, supervision and mentoring, and manuscript development. ML-J co-led the development and the implementation of the training program, contributed to the selection, recruitment, training and supervision of therapists, and the development of the manuscript. RW contributed to therapist recruitment, training and further development of the training, adherence monitoring, supervision and mentoring program, evaluation of the training program, and the development of the manuscript. EM led the development of the therapeutic model, and the design and the implementation of the training program and also contributed to the evaluation of the survey results and development of the manuscript. All authors contributed to the article and approved the submitted version.

## Conflict of Interest

ST, WR, PG, FR, BR, R-LJ, and EN have received consulting fees and salary support from COMPASS Pathways PLC. EN is an owner of Fluence, an advisor to Sansero Life Sciences, independent contractor researcher for Multidisciplinary Association for Psychedelic Studies Public Benefit Corporation (MPBC), and former staff clinical researcher at NYU School of Medicine on research projects funded by Heffter Research Institute, MPBC, and Riverstyx Foundation. ML-J, RW, and EM are employees of COMPASS Pathways PLC.

## Footnotes

^1^https://mapspublicbenefit.com/training

^2^https://www.ciis.edu/research-centers/center-for-psychedelic-therapies-and-research/about-the-certificate-in-psychedelic-assisted-therapies-and-research

^3^https://www.fluence8.com/postgraduate-certificate-in-psychedelic-integration-therapy

## Abbreviations

DMT: N,N-Dimethyltryptamine
FDA: United States Food and Drug Administration
GCP: Good Clinical Practice
LSD: Lysergic acid diethylamide
MDMA: 3,4 methylenedioxy-methamphetamine
TRD: Treatment-resistant depression.
