# Supplementary material for: Development and Evaluation of a Therapist Training Program for Psilocybin Therapy for Treatment-Resistant Depression in Clinical Research
Source: Front Psychiatry. 2021 Feb 3;12:586682. doi: 10.3389/fpsyt.2021.586682 (PMC7908919; doi:10.3389/fpsyt.2021.586682)
Supplement: Supplementary file 1 [file Data_Sheet_1.pdf]

COMPASSION
Navigating Mental Health Pathways

Therapist Code of Ethics and Conduct

Psilocybin and similar psychoactive compounds are known to facilitate profound experiences including non-ordinary states of consciousness. These experiences, while thought to be therapeutic and beneficial for recovery and personal growth, may put patients in a vulnerable state not only during the experience but afterwards as well.

The safety and benefits of a psilocybin session and integration of the experience depend largely on the trust or therapeutic alliance between a patient and a therapist. Both patient and therapist enter a shared agreement on rules of behaviour for the entire study.

As a therapist working at COMPASS Pathways trial sites, I will:

Safety and compassion

- Hold patient safety as a priority
- Protect each participant's emotional and physical health and safety throughout the treatment, including periods of preparation, session and integration
- Recognise my own limitations of knowledge, understanding and experience, and seek advice, when needed, to deliver high quality, safe, and compassionate care
- Conduct sessions in ways that cultivate awareness, empathy, and respect throughout the therapeutic process

Honesty, integrity, and self-responsibility

- Be aware of how my own belief systems, values, needs, and limitations affect my care for my patients
- Remember that during psilocybin sessions, participants may be especially vulnerable and susceptible to suggestion and manipulation; practise with integrity, protect patients, and prevent anyone from using that vulnerability in ways that might harm patients or others
- Be trustworthy, responsible, and accountable for my actions, and conscious of how these actions impact patients and colleagues
- Deliver care only within the scope of practice as determined by education and training

Equality, inclusion, and diversity

- Uphold and practice equal treatment of patients and colleagues regardless of their age, disability, sexual orientation, sex, race, religion and/or beliefs
- Treat people as individuals and respect their individual identities; respect personal and professional boundaries

Confidentiality

- Maintain and safeguard the confidentiality of all patient, potential patient, and employee information
- Ensure that personal and other sensitive information is held securely and shared appropriately; refrain from disclosing information to any unauthorised third party

and from using any documents, data, or information obtained in the course of employment for any unauthorised purpose

Therapeutic alliance and boundaries

- Build professional and compassionate therapeutic alliances with patients
- Bolster therapeutic alliances with patients by establishing and maintaining healthy emotional and physical boundaries. Make clear and agree upon the behavioural boundaries for participants and colleagues in advance of any session
- Recognise that insights gained from psychedelic sessions can catalyse desire for personal and social change; use special care to help direct the energies of those I support, as well as my own, in responsible and balanced ways
- Follow professional ethical guidelines. Financial, transactional, romantic and/or sexual relationships or interactions between therapists and patients are strictly prohibited both during and after the study period. Any failure to comply with boundary specifications will result in termination of the working relationship between the therapist and COMPASS Pathways, and could result in serious professional consequences

Therapist name and site
Date and signature
